# Establishment and Characterisation of Heterotopic Patient-Derived Xenografts for Glioblastoma

**DOI:** 10.3390/cancers12040871

**Published:** 2020-04-03

**Authors:** Sarah Meneceur, Annett Linge, Matthias Meinhardt, Sandra Hering, Steffen Löck, Rebecca Bütof, Dietmar Krex, Gabriele Schackert, Achim Temme, Michael Baumann, Mechthild Krause, Cläre von Neubeck

**Affiliations:** 1 OncoRay–National Center for Radiation Research in Oncology, Faculty of Medicine and University Hospital Carl Gustav Carus, Technische Universität Dresden, Helmholtz- Zentrum Dresden-Rossendorf, 01307 Dresden, Germany; annett.linge@uniklinikum-dresden.de (A.L.); steffen.loeck@oncoray.de (S.L.); rebecca.buetof@uniklinikum-dresden.de (R.B.); Michael.Baumann@dkfz.de (M.B.); Mechthild.Krause@uniklinikum-dresden.de (M.K.); claere.vonneubeck@uk-essen.de (C.v.N.); 2 Helmholtz-Zentrum Dresden-Rossendorf, Institute of Radiooncology–OncoRay, 01307 Dresden, Germany; 3 German Cancer Consortium (DKTK), Partner Site Dresden, 01307 Dresden, Germany; gabriele.schackert@uniklinikum-dresden.de (G.S.); Achim.Temme@uniklinikum-dresden.de (A.T.); 4 German Cancer Research Center (DKFZ), 69120 Heidelberg, Germany; 5 Department of Radiotherapy and Radiation Oncology, Faculty of Medicine and University Hospital Carl Gustav Carus, Technische Universität Dresden, 01307 Dresden, Germany; 6 National Center for Tumour Diseases (NCT), Partner Site Dresden, 01307 Dresden, Germany; 7 Institute for Pathology, Faculty of Medicine and University Hospital Carl Gustav Carus, Technische Universität, 01307 Dresden, Germany; matthias.meinhardt@uniklinikum-dresden.de; 8 Institute for Legal Medicine, Faculty of Medicine and University Hospital Carl Gustav Carus, Technische Universität, 01307 Dresden, Germany; sandra.hering@tu-dresden.de; 9 Department of Neurosurgery, Medical Faculty and University Hospital Carl Gustav Carus, 01307 Dresden, Germany; Dietmar.Krex@uniklinikum-dresden.de; 10 Department of Particle Therapy, University Hospital Essen, University of Duisburg-Essen, 45147 Essen, Germany

**Keywords:** patient-derived xenografts, preclinical models, cancer stem cell markers, glioblastoma, growth data

## Abstract

Glioblastoma is an aggressive brain tumour with a patient median survival of approximately 14 months. The development of innovative treatment strategies to increase the life span and quality of life of patients is hence essential. This requires the use of appropriate glioblastoma models for preclinical testing, which faithfully reflect human cancers. The aim of this study was to establish glioblastoma patient-derived xenografts (PDXs) by heterotopic transplantation of tumour pieces in the axillae of NMRI nude mice. Ten out of 22 patients’ samples gave rise to tumours in mice. Their human origin was confirmed by microsatellite analyses, though minor changes were observed. The glioblastoma nature of the PDXs was corroborated by pathological evaluation. Latency times spanned from 48.5 to 370.5 days in the first generation. Growth curve analyses revealed an increase in the growth rate with increasing passages. The methylation status of the *MGMT* promoter in the primary material was maintained in the PDXs. However, a trend towards a more methylated pattern could be found. A correlation was observed between the take in mice and the proportion of Sox2^+^ cells (*r* = 0.49, *p* = 0.016) and nestin^+^ cells (*r* = 0.55, *p* = 0.007). Our results show that many PDXs maintain key features of the patients’ samples they derive from. They could thus be used as preclinical models to test new therapies and biomarkers.

## 1. Introduction

Worldwide, approximately 300,000 new cases of malignant tumours in the brain and nervous system were diagnosed in 2018, representing 1.6% of all cancers [1]. Among the tumours of the central nervous system, glioblastoma is the most common (49%) malignancy according to the World Health Organisation. Glioblastoma are grade IV tumours and are characterised by a highly aggressive and invasive phenotype, resulting in a very low 5-year survival of 6% [2]. The standard treatment for glioblastoma patients (*Stupp* regime) consists of surgery, radiotherapy (RT) and concurrent followed by adjuvant temozolomide [3,4]. The alkylating agent temozolomide in combination with RT was shown to prolong survival and increase median survival to 14.6 months, in comparison to 12.1 months with RT alone [3,5]. (Hyper-)methylation of the *MGMT* promoter (O6-Methylguanin DNA-Methyltransferase), a gene encoding a DNA repair protein removing alkyl adducts, constitutes a favourable factor in glioblastoma response to temozolomide [6]. However, most patients are currently treated according to the *Stupp* regime in the absence of alternative treatment options.

Resistance to treatment and tumour development have been attributed to a subpopulation of cancer stem cells (CSC), which are able to proliferate and reconstitute the heterogeneity of the tumour mass [7,8,9,10]. Different markers have been described and have been associated with the stemness ability of the cells [11]. Among these markers, Sox2 is a transcription factor expressed in embryonic stem cells and in proliferative regions of the adult brain. It was shown to be overexpressed in glioblastoma, and studies suggest Sox2 as a prognostic factor for glioblastoma [12]. The class VI intermediate filament nestin is also discussed as an overexpressed cancer stem cell marker in glioblastoma [13]. However, nestin is also present in endothelial cells. CD95 is involved in apoptosis as well as the invasive phenotype of glioblastoma cells and was shown to be a prognostic factor in glioblastoma, as well as a biomarker for stem cell ability [14,15].

The prognosis for glioblastoma patients is particularly poor, which emphasises the need for a better understanding of the pathogenesis of glioblastoma, along with the development and validation of new treatment strategies.

For that purpose, mouse preclinical models are of major relevance. In glioblastoma, heterotopic and orthotopic tumour models originating from established cell cultures and from patients’ samples have been described [16,17,18]. Cell-line-derived xenografts (CDXs) have been proposed as preclinical models for glioblastoma. However, two main limitations arise from them. (1) their response to therapy might not mirror the clinical situation. In radiotherapy, glioblastoma CDXs have been reported to show a lower radioresistance in comparison to the clinical situation [19,20]. (2) glioblastoma are characterised by a high intra-tumoural heterogeneity [21], which is likely not recapitulated in the monoclonal CDXs.

Patient-derived xenografts (PDXs) have been shown to resemble human tumours, which suggests them as appropriate models for preclinical studies (reviewed in [22]).

In the present study, we aimed at generating a panel of glioblastoma PDX models to test new therapies in combination with RT. Resemblance with patient material was directly assessed by histopathological analyses, immunohistochemical analyses of putative cancer stem cell (CSC) markers and methylation-specific PCR of the *MGMT* promoter (MSP).

## 2. Results

### 2.1. Presentation of the Patients’ Cohort

Twenty-two patients diagnosed with primary glioblastoma were recruited for this study (50% female and 50% male), with informed consent. Their age ranged from 53 to 85 years at diagnosis (Table 1). The methylation of the *MGMT* promoter was assessed as part of the routine pathological work-up: 45.5% of the tumours were categorised as *MGMT*-methylated, while 54.5 % were stated as non-methylated.

### 2.2. Take Rate in Nude Mouse and Tumour Measurements

To preserve tumoural architecture and microenvironment, samples were transplanted as tumour chunks on the nude mice. A total of 10 out of the 22 samples transplanted resulted in at least one growing tumour in the nude mouse, which corresponds to a take rate of 46%. For each specimen, 5 to 10 samples were transplanted with a success rate ranging from 10% to 70% (Table 2).

**Figure 1:**
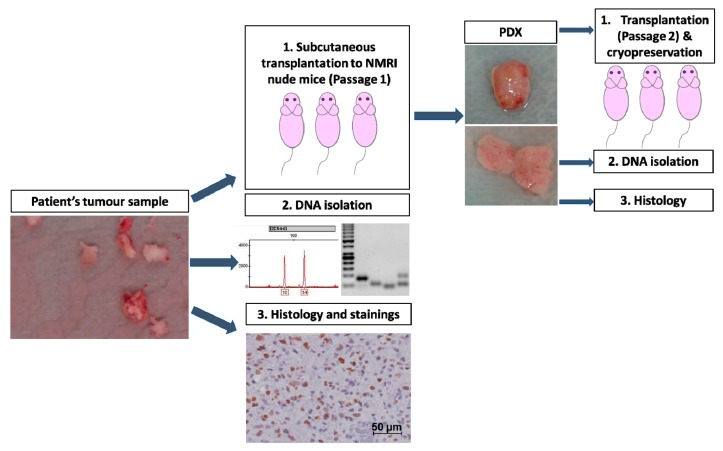
Experimental design for the establishment. Patients’ samples were obtained from the neurosurgery department of the Medical Faculty and University Hospital Carl Gustav Carus, Dresden, Germany. Blood and necrotic tissue were removed from the sample, and tumour chunks were prepared for transplantation. One sample was transplanted into each axilla of the mouse. Growing tumours were excised and (**1**) conserved for further transplantation (cryopreservation and direct transplantation); (**2**) stored in liquid nitrogen for e.g., DNA isolation; (**3**) FFPE fixed for e.g., histological analysis.

**Figure 2:**
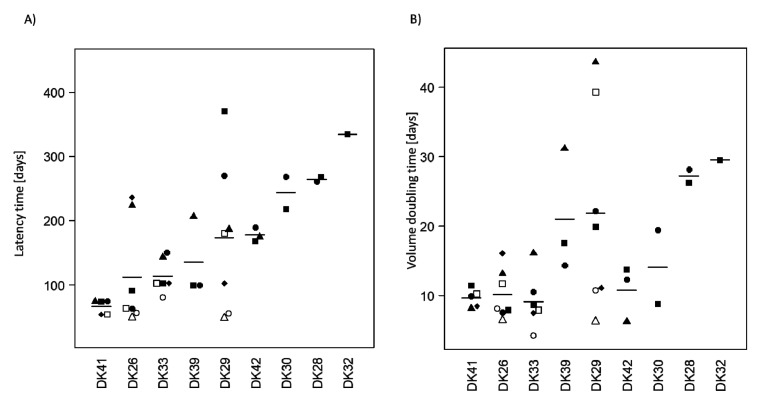
Growth parameters in the first-generation glioblastoma PDXs. (**A**) The latency time is defined as the time after transplantation until observation of a 40 mm^3^ tumour. (**B**) The volume doubling time (VDT) was estimated for each first-generation PDX; the mean value is represented for each model. The latency time and the VDT correlate (*r* = 0.67, *p* < 0.0001). Models are sorted by increasing mean latency times. Each symbol represents a PDX in the respective model. DK35: data not available as the tumour did not reach the required size for calculation.

**Figure 3:**
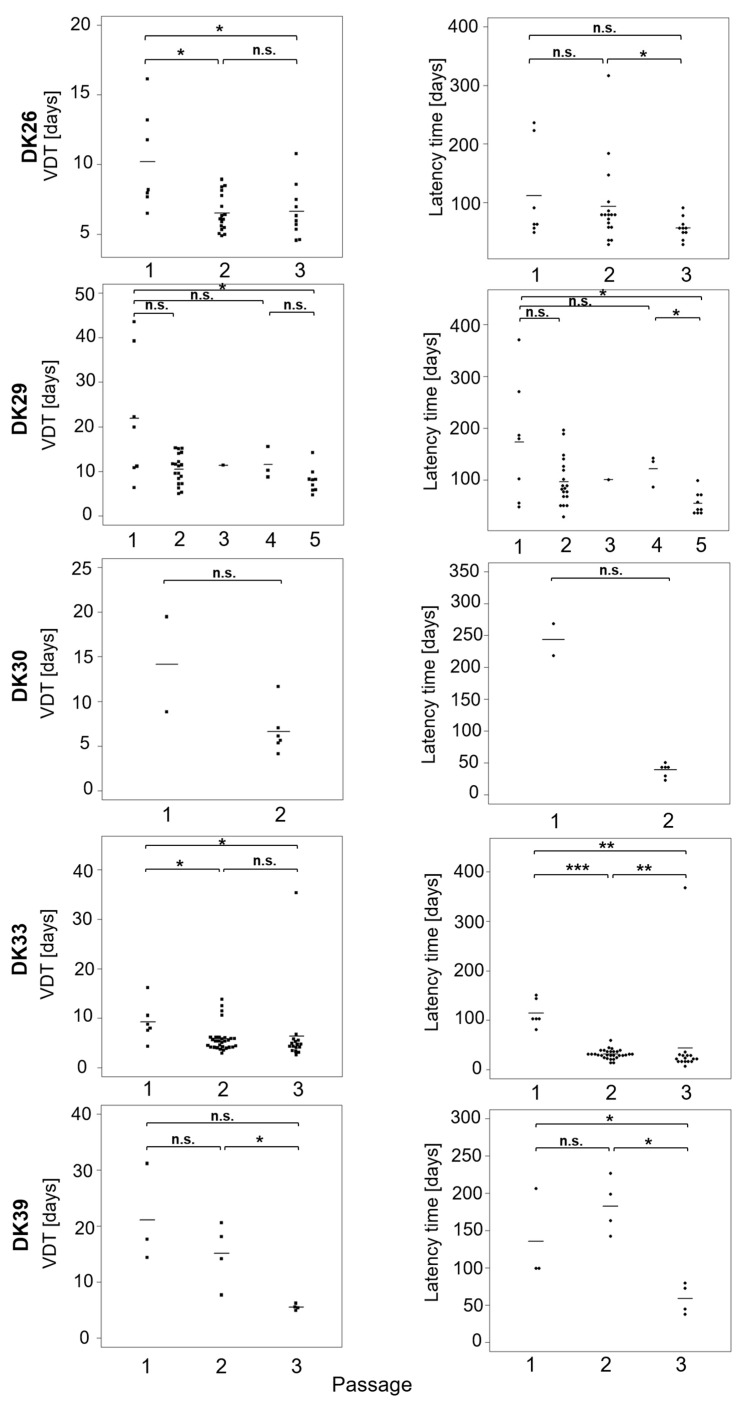
Volume doubling time and latency time in the subsequent passages. For each passage, the VDT was compared with the VDT of the first passage and the previous passage. The *y*-axis scales are different. Comparisons were performed with the Mann–Whitney test n.s. non-significant; * *p* < 0.05; **: *p* < 0.01; ***: *p* < 0.001.

Tumours were passaged up to five times to establish a tumour line (Figure 1). The take rate at the subsequent passages are presented in supplementary Table S1. Microsatellite analysis confirmed the human origin of the tumours, though minor changes (loss of heterozygosity, imbalance) could be observed in all models (Figure S1). The latency time to observe a growing tumour ranged from 48.5 days to 370.5 days with a mean of 147 days (Figure 2A). In many cases, a decrease in the latency times could be observed in the subsequent passages, underlining a potential increase in the aggressiveness of the tumour and a better adaptation to the murine host (Figure 3). The average volume doubling time (VDT) was estimated to be 15 days, with values ranging from 4 days in DK33 to 44 days in DK29 (Figure 2B).

A decrease in the VDT could be observed in subsequent passages, resonating with the observations on latency times (Figure 3). A significant correlation (Spearman) was observed between the VDT and the latency time (*r* = 0.67, *p* < 0.0001). These data suggest that, in some cases, the tumours in the next generation grow faster than in the first passage.

**Figure 4:**
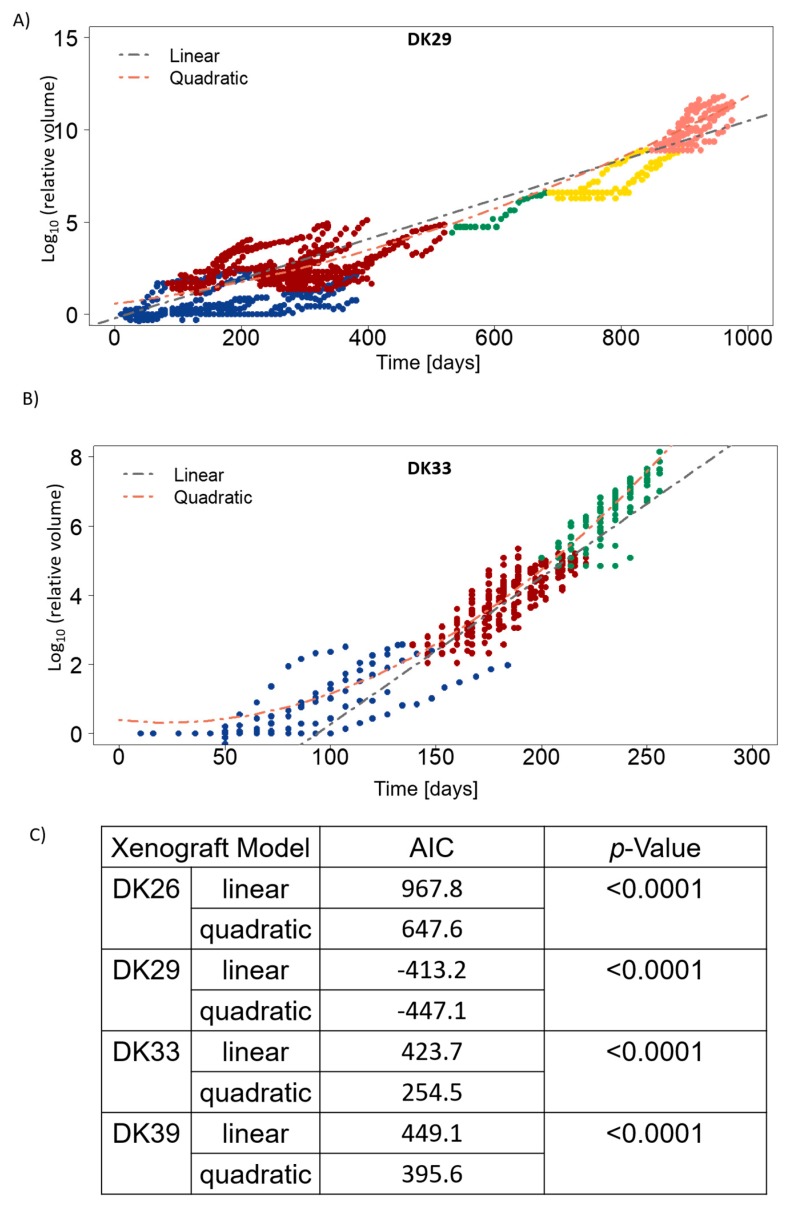
The growth rate of the PDXs increases over time. (**A**) Cumulative growth curve in DK29, which was serially transplanted for five passages. (**B**) Cumulative growth curve in DK33, which was serially transplanted for three passages. The linear and quadratic model fits are represented as dotted lines. Each dot represents a tumour measurement of a growing tumour. Blue: passage one; red: passage two; green: passage three; yellow: passage four; pink: passage five. (**C**) Results of the ANOVA analysis, which was used to compare the two models (linear and quadratic) with R. AIC: Akaike Information Criterion. The model with the lowest AIC is considered the best-fitting model.

To test this hypothesis, cumulative growth curves were calculated according to [24] (Figure 4, Figure S2). The lineage of the tumours was recorded, and one growth curve was established from all the tumours at the different passages. Linear and quadratic models were fitted and compared (Figure 4C). The significantly decreasing AIC (Akaike Information Criterion) values for the quadratic models suggests that the growth rate increases with time.

### 2.3. Histology and Putative Stem Cell Markers

**Figure 5:**
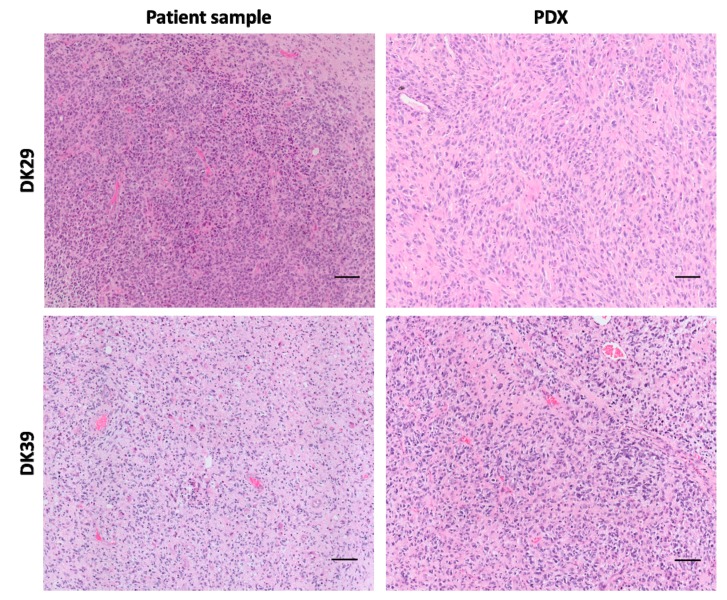
Hematoxylin and eosin stainings of the patient sample and the corresponding PDX. Scale bar = 100 µm.

Neuropathological evaluation confirmed (a) the glioblastoma nature of the tumours grown in mice and (b) a high similarity between the primary sample and the first-generation PDX in most models. PDXs conserve characteristic pathological features of glioblastoma, namely cell pleomorphism, a high cell density, endothelial hyperproliferation and pseudopalisading (Figure 5, Figure S3). Besides, PDXs present a high proportion of tumour cells in comparison to immune cells, endothelial cells and connective tissue (Figure S4).

**Figure 6:**
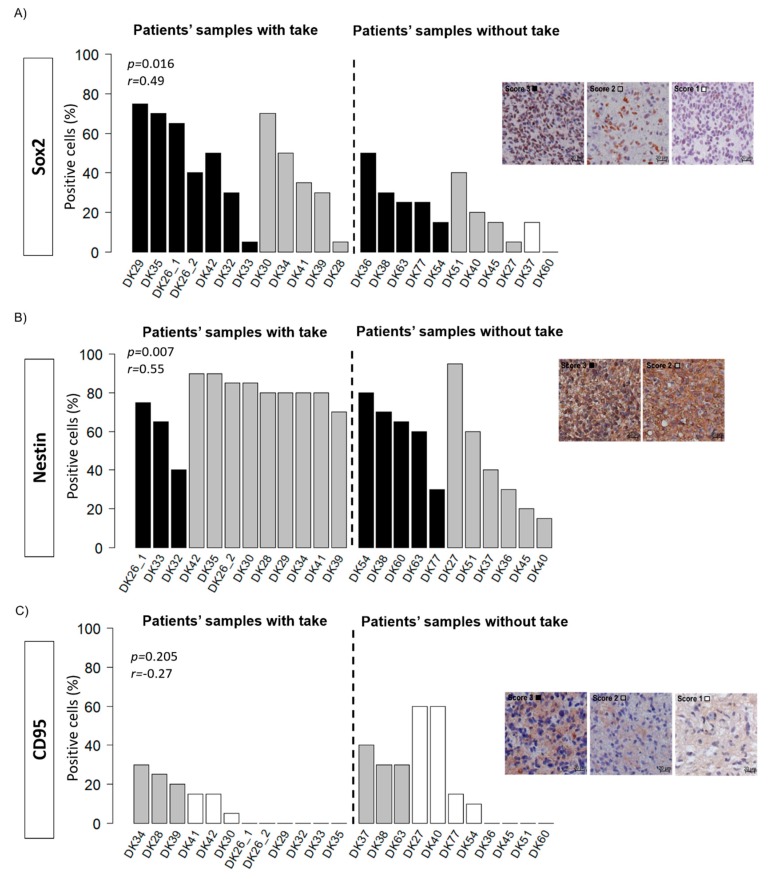
Putative cancer stem cell markers in the patient material. (**A**) The stainings were evaluated by two independent observers. (**B**) The proportion of positive cells (0–100%) and the intensity of the staining (0–3) were assessed (negative: intensity score 0, white: intensity score 1, grey: intensity score 2, black: intensity score 3). (**C**) Association between the proportion of positive cells and the take in the first generation were analysed; results (*p*-value and coefficient of correlation (Spearman)) are presented in the graph.

**Figure 7:**
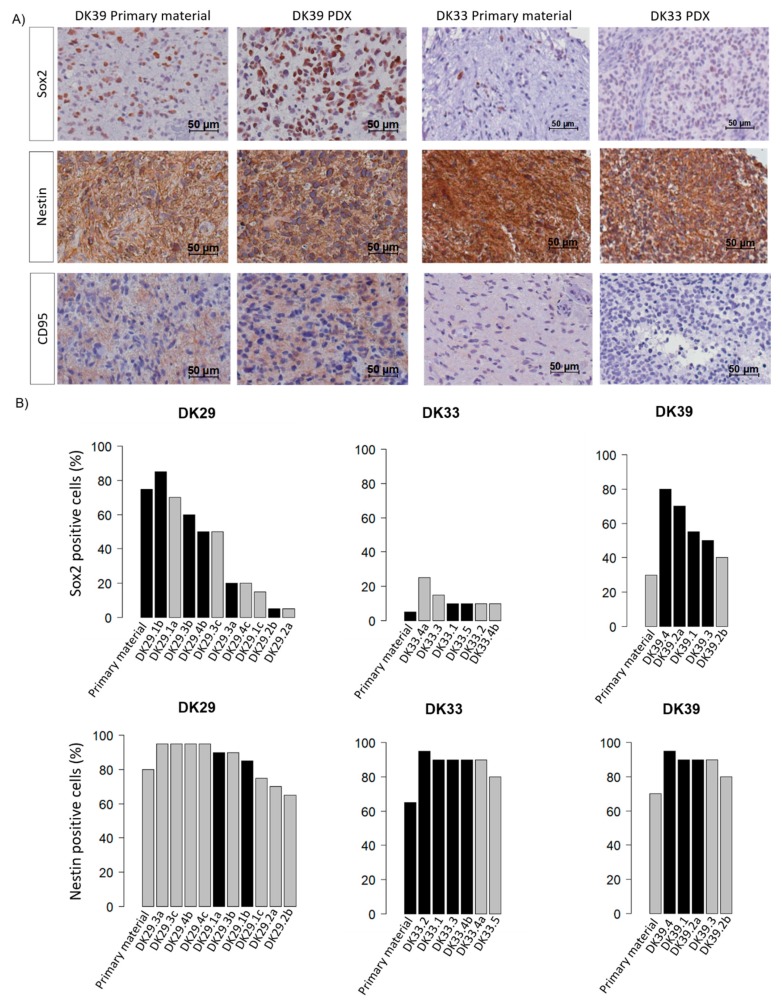
Comparison of the putative cancer stem cell markers in the patients’ samples and in the first-generation PDXs. The stainings were evaluated by two independent observers. The proportion of positive cells (0–100%) and the intensity of the staining (1–3) were assessed (white: intensity score 1, grey: intensity score 2, black: intensity score 3). (**A**) Immunohistochemistry images (Sox2, nestin, CD95) for two representative patients’ samples (DK39 and DK33) and their respective PDXs. (**B**) Bar graphs presenting the proportion of positive cells and the intensity of staining in the patients’ samples and the derived PDXs in DK29, DK33, and DK39.

According to the CSC hypothesis, the uncontrolled proliferation of a subpopulation of cells with stem cell abilities leads to the development of tumours. It was hypothesised that the ability of the tumour samples to grow in the mice was linked to the amount of CSC in the primary material. To test this hypothesis, the expression of Sox2, nestin, and CD95 was assessed in the patient material (Figure 6). In the primary material, Sox2 showed a wide range of expression levels with 80% positive cells in DK29 and negative samples in DK50. As for nestin, a high staining intensity was found in all the samples, with proportions of positive cells ranging from 15% to 95%. A moderate correlation (Spearman) was found between the proportion of Sox2^+^ (*r* = 0.49, *p* = 0.016) and nestin^+^ cells (*r* = 0.55, *p* = 0.007) and the take in mice. The expression of CD95, however, was low with 13 out of 22 samples positive and positivity levels not exceeding 60%. Its expression was not statistically associated with the take in mice. The first generation of xenografts were stained for the same markers to assess the degree of similarities between the primary material and the PDXs (Figure 7). Generally, the expression of Sox2 and nestin was conserved in the PDXs. However, DK29 and DK41 showed few discrepancies comparing the original material and the PDXs (Figure 7, Figure S5). This observation suggests a certain heterogeneity in the obtained models, which might be of interest when testing new therapeutics. The expression of CD95 was also low in the PDXs (Figure S6).

### 2.4. Methylation of the MGMT Promoter

**Figure 8:**
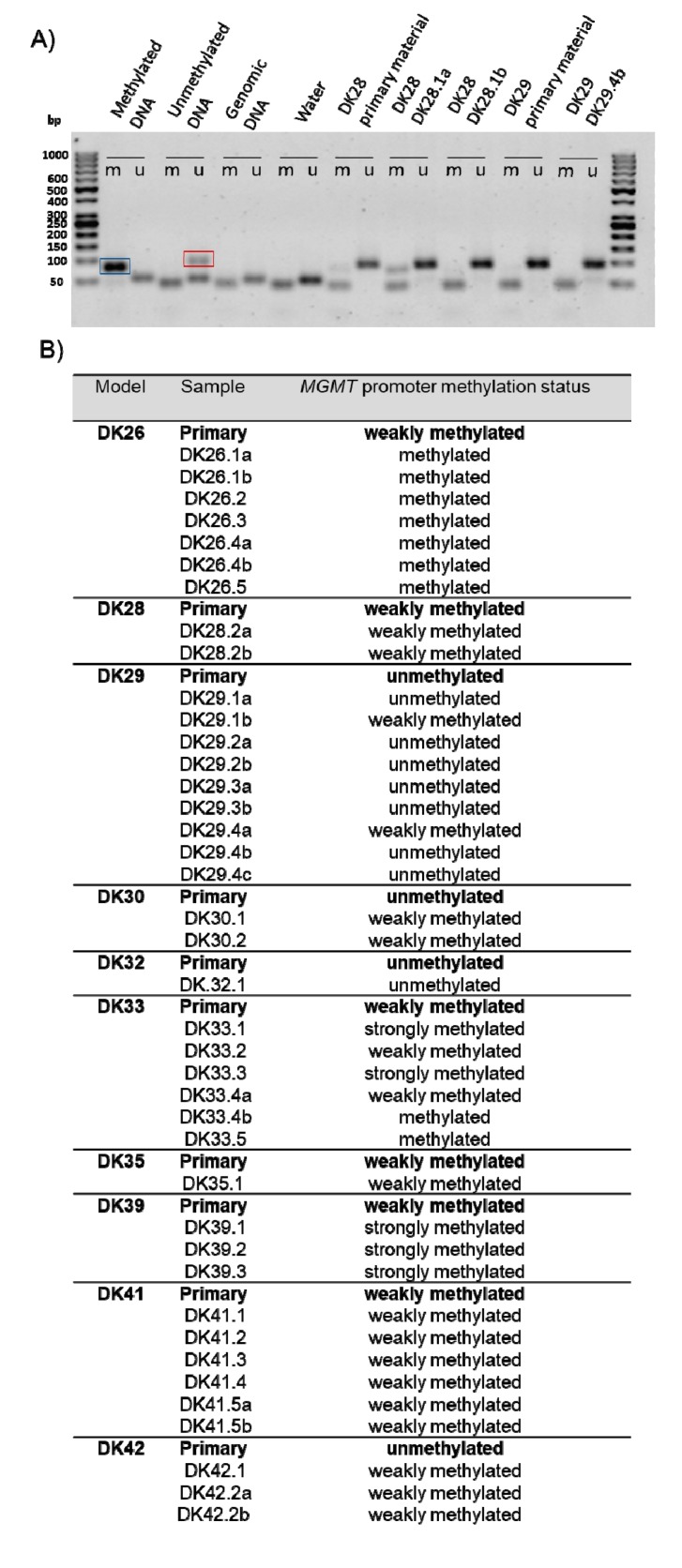
Semi-quantitative analysis of the *MGMT* promoter methylation. (**A**) Representative agarose gel with methylation-specific PCR (MSP). Blue square: fragment resulting from the amplification of a methylated template (M); red square: fragment resulting from the amplification of an unmethylated template (U). (**B**) Summary table of the semi-quantitative analysis of the MSP. For each sample, the M/U ratio was calculated. Samples were classified as unmethylated (M = 0), weakly methylated (0 <M/U< 1), and strongly methylated (M/U > 1). Samples with U = 0 were considered methylated.

The methylation status of the *MGMT* promoter was analysed in the primary material and in the PDXs by methylation-specific (MSP) (Figure 8A). Qualitative analyses revealed that 17 samples present evidence of methylation on the *MGMT* promoter, with the exception of DK29, DK30, DK32, DK42, and DK54. These results do not completely match the information from clinical testing, which may potentially be attributed to the different method used to assess methylation (sensitivity and threshold) and the intra-tumoural heterogeneity of the samples used for analysis. No statistical association between the methylation status of the *MGMT* promoter (clinical information) and observed tumour take in mice was found. The patient material and the PDXs were classified as unmethylated, weakly methylated and strongly methylated (Figure 8B). A shift towards a higher methylation status was detected in four tumour lines (DK26, DK30, DK39, DK42) (Figure 8B). In four cases (DK28, DK32, DK35, DK41), the methylation status was conserved in all the PDXs. Finally, in two cases (DK29 and DK33), a heterogeneity in the different PDXs deriving from the same material was detected: while some PDXs maintained the same pattern as the primary material (DK29 unmethylated, DK33 weakly methylated), others showed a higher methylation status. The methylation status did not decrease in the PDXs.

## 3. Discussion

Glioblastoma is associated with a poor survival, attributed to the low sensitivity to current treatment. To develop new therapies and increase the survival rates, a better understanding of the particular tumour biology of glioblastoma is essential. This requires the development of suitable glioblastoma tumour models that properly reflect the aggressiveness of glioblastoma observed in the clinics and recapitulate the key characteristics of the disease.

In the present study, we describe the successful engraftment of glioblastoma samples to nude mice, to generate heterotopic PDXs. Other studies reported successful take rates of 22–26% for glioblastoma in nude mice [25,26]. In our study, the subcutaneous heterotopic transplantation of tumour chunks on NMRI nude mice yielded 46% success. However, we observed inter-patient variation of 10–70% take for the primary material. This could be attributed to the heterogeneous nature of glioblastoma, representing different amounts of cancer-initiating cells and abilities to proliferate. It was previously reported that within the same glioblastoma tumour, differences in the resulting model such as tumourigenicity could be observed depending on the site where the tumour was harvested (e.g., rim, center) [27]. In our study, no information on the exact location of the tumour chunk was available. The differences in growth properties can be attributed to cell viability, necrotic area and CSC population. Tumour samples were indeed transplanted as pieces, whose composition is likely heterogeneous, without prior cell dissociation to maintain the architecture of the tissue. In contrast, the use of cell suspension and cell dissociation leads to the disruption of interactions between cells, which might affect engraftment success of the sample ([28]; reviewed in [29,30]).

Tumour samples were subcutaneously transplanted to the axillae of the mice, which corresponded to a non-natural microenvironment for the tumour. The brain tumours’ microenvironment was shown to be complex and encompassed various cell types (e.g., endothelial cells, immune cells such as macrophages) as well as cross talks with brain cells (neurons, astrocytes) (reviewed in [31]). Orthotopic transplantation of glioblastoma cell suspension provides a microenvironment which closely resembles the human situation. They were shown to retain glioblastoma properties [18]. However, their establishment is labour intensive, as it requires microsurgery and imaging modalities such as MRI to follow tumour growth. As a consequence, less mice can be handled in an experiment. Furthermore, the growing tumours need to be excised at low tumour diameter upon neurological symptoms to ensure minimal burden to the animal, and mice must be sacrificed. Nevertheless, they can be highly relevant to test therapies which target processes such as migration and the invasion potential of glioblastoma. In our study, the samples were transplanted heterotopically, which enables easy monitoring and access to the tumour notably for radiotherapy experiments, high numbers of transplanted animals in parallel and sufficient tumour material for the establishment of models. In this study, PDX tumours were also cryopreserved in DMSO-containing medium, to be thawed and re-engrafted for future experiments. Besides the nature of cryoprotectant, different factors can influence the recovery of tumour growth after cryopreservation, such as the time lapse between removal of the sample from blood supply and cryopreservation, presence of necrotic tissue in the sample, the size of the frozen sample and its preservation, and the washing off of cryoprotectant before re-engraftment [32]. Studies about the transplantation of tumour samples after cryopreservation report different recovery rate of PDXs after cryopreservation (22% [26] (glioblastoma); 50% [33] (lung cancer), 100% [34] (liver metastases of uveal melanoma)). Additionally, Kageyama et al. showed that PDXs derived from frozen tissue maintained copy number variation (CNV) and proteomic profiles, but presented slower growth compared to the transplantation of fresh tissues.

Subpopulations of CSC have been described in glioblastoma and are characterised by the expression of different markers ([7,8]; reviewed in [35]). Here, the expression patterns of three CSC markers (Sox2, nestin, CD95) were investigated in the primary patient materials and in PDXs. The proportion of cells expressing Sox2 spans from 0% to 75%. This observation is consistent with previous reports [36,37], which showed that the expression of Sox2 was not limited to a small subset of cells but was more widespread in the tumour [37]. Sox2 is reported to be involved in the regulation of genes associated with malignancy and the maintenance of cell plasticity. Besides, we found a moderate correlation between the proportion of Sox2^+^ cells and tumour take in mice, pinpointing an association between Sox2 expression and tumourigenicity of transplants. In first-generation PDXs, the expression of Sox2 is maintained and appears to recapitulate the pattern observed in the patient samples. Further, a high proportion of nestin^+^ cells was observed in the primary material, which is consistent with other reports [13,38,39]. A moderate positive correlation between the proportion of positive cells and the engraftment in the mice was observed. The prognostic value of nestin is controversial [40]. It was found to be associated with the malignancy of gliomas [38], while others failed to show any correlation between nestin expression and the prognosis in glioblastoma [41]. The nestin expression pattern is conserved in PDXs and >60% cells were positive in all the PDXs. This observation suggests that PDXs might be enriched in cancer-initiating cells, which might explain their ability to grow in the host and to be perpetuated over several generations. Similar expression patterns in primary patient samples and PDXs also point towards a possible function of the filament protein nestin in engraftment. Although the functional role of nestin in malignancy is poorly understood [39], a study in glioblastoma cell lines suggests a role for nestin in tumourigenesis [42].

In contrast to Sox2 and nestin, the expression of CD95 was low in the cohort. Thirteen primary samples were positive for CD95. The proportion of positive cells was <60%. For the PDXs, only five models expressed this marker. No correlation with the take rate was found and the function of CD95 as a CSC marker of glioblastoma appears questionable. Other markers (e.g., CD133, CD44) have been shown to be associated with stem cell ability in glioblastoma (reviewed in [35]), and it would be interesting to evaluate their expression patterns in PDXs.

Microsatellite analysis corroborated that the PDXs originated from the transplanted material, although minor changes, such as loss of heterozygosity and imbalance could be observed. Other studies described genetic modifications (e.g., gene amplification, gain/loss of mutation) in early passage PDXs in comparison to the patients’ samples [26,43], despite a high similarity. Some changes might be occurring *de novo*, or they might reflect selection of pre-existing subpopulations during transplantation.

The latency time for tumour growth exceeds 1.5 months. This precludes the use of these models to test therapies for a specific patient and provide real time treatment recommendation. However, the models could be used to study alternative therapies in comparison to standard treatments and might help in the future to guide more personalised treatment decisions. They could hence be used to test the efficiency of new drugs, combined treatments or sensitising agents (e.g., radiotherapy, chemotherapy, inhibitors). For this purpose, these models could be transplanted orthotopically, to account for the blood–brain barrier, which despite being disrupted in gliomas, might limit efficiency of therapeutic molecules (reviewed in [44,45]). For radiotherapy experiments, it would be interesting to evaluate their response to ionising radiation by performing tumour control dose 50 experiments [46,47]. Additionally, these models could be used for discovery and validation of biomarkers for radiotherapy efficiency, which are essential to stratify patients and allow treatment individualisation [18,48,49].

In the subsequent passages, a significant reduction of the latency times and VDT was observed. Cumulative growth curves were established for the tumour lines that had been passaged more than twice. In all the cases, the linear quadratic model fitted the data better than the linear model. This suggests that the growth rate of glioblastoma PDXs increases with the passages in glioblastoma, which is in line with other studies [24]. Altogether, these results suggest a better adaptation to the murine host, a potentially higher aggressiveness and clonal selection process of the tumour in subsequent models, which might be associated with a higher divergence from the human tumour. Nonetheless, histological observation confirms that tumours of higher passages conserve glioblastoma features. The genome of PDXs was shown to be representative of primary human tumours [18,50,51]. Gene expression analyses also indicate a high similarity between PDXs and the patient sample they derive from [48,50,52]. Additionally, a recent study by whole-exome sequencing in glioblastoma and their parent tumours showed that most genetic driver mutations are conserved in PDXs, although gain and loss were also observed, indicating potential clonal selection [53]. It can be speculated that with increasing passage, the number of genetic and transcriptomic alterations will increase in the specific mouse microenvironment. Although some studies indicate that PDXs conserve a stable genome, proteome, and transcriptome over passaging [50,54,55,56], data pointing towards a shift from the original primary tumour upon xenografting have been reported (copy number alterations, [51,57]). Interestingly, it was found that copy number alterations in brain tumours are the most stable among the tested 1100 PDXs across 24 cancer entities [51]. Drift from the original tumour can notably be attributed to the lack of human stromal component, which is replaced by murine cells [52,55]. Further genetic and epigenetic analyses are however needed to elucidate the question of glioblastoma heterogeneity and adaptation to the host.

The methylation status of the *MGMT* promoter was investigated in the patients’ samples and in the PDXs by MSP. In the PDXs, a methylation profile comparable to the respective primary patients’ samples was observed. However, a trend towards a more methylated profile can be observed in 52.5% of the samples. This suggests a different clonal selection upon engraftment potentially associated with different CSC populations. The hypermethylation process might also potentially be attributed to the new microenvironment. Hypermethylation has also been observed in cell lines and was attributed to an active epigenetic change due to the culture conditions [58,59,60].

## 4. Materials and Methods

### 4.1. Human Tumour Samples

The generation of experimental tumour models from patient material has been approved by the local ethics committee (EK152052013, EK3231122008). Glioblastoma specimens were obtained after resection from the Department of Neurosurgery, Faculty of Medicine and University Hospital Carl Gustav Carus, Technische Universität Dresden, Germany with patients’ written informed consent. Patients recruited for this study did not receive any treatment before surgery. Patient’ characteristics are presented in Table 1.

A pseudonym was attributed to each patient and clinical information was collected in the RadPlanBio database [23] for long term follow-up.

After receipt in DMEM (Dulbecco’s modified Eagle’s medium, Biochrom GmbH, Berlin, Germany), the material was processed within an hour under a laminar flow (removal of necrotic tissue and blood). The tissue was rinsed in NaCl (NaCl, Fresenius Kabi, Germany GmbH), and cut into small pieces for transplantation in the mice.

### 4.2. Animals

Seven to fourteen-week-old male and female NMRI (nu/nu) mice were used. The mice were maintained in a pathogen-free animal breeding facility (OncoRay, Medical Faculty Carl Gustav Carus, Technische Universität Dresden, Germany) with a cycle of 12 h of light and 12h of dark and were fed *ad libitum*. The animal facilities and the experiments were approved according to institutional guidelines and German animal welfare regulations (AZ 24-9168.11-1/2012-52 “Establishment of tumour models from patients’ biopsies for experimental radiation biology and radiooncological evaluation in animal models”). For further immunosuppression, the animals received whole-body irradiation with 4 Gy 1–4 days prior to tumour transplantations. All irradiations were performed with 200 kV X-rays (Isovolt 320/13, Seifert, Ahrensdorf, Germany; 20 mA; 0.5 mm Cu filter; dose rate 1 Gy/min). After the whole-body irradiation, antibiotics were added to the drinking water for 5 days to prevent infection. The design of the study is presented in Figure 1. Patient-derived material was transplanted in the axillae of 5–10 mice depending on the amount of material provided. Due to the soft tissue texture of glioblastoma, the transplantation of one tumour piece could result in the growth of several tumours at one transplantation site. Only the largest nodule was analysed for take rate, latency times and growth. The smaller nodules were however excised and analysed when the material was sufficient. Tumour growth was monitored weekly with a calliper. PDX tumours were excised without treatment when they were closed to reaching end size (200 mm^3^) or when the mice showed worsening health conditions. First-generation PDXs were formalin-fixed and paraffin-embedded for histology, flash-frozen for DNA isolation, cut in pieces for further transplantation, and cryopreserved in DMEM (10% DMSO) for future transplantation. PDXs were passaged and maintained on 1 to 5 generations. Tumour samples were placed in a freezing container at −80 °C for a controlled cooling rate of −1 °C/minute and subsequently kept in liquid nitrogen for long term storage.

### 4.3. Stainings

#### 4.3.1. Hematoxylin and Eosin

Tumour samples were fixed in formalin (4% formaldehyde solution, neutral, buffered, SAV liquid production GmbH, Flintsbach am Inn, Germany) and embedded in paraffin (Engelbrecht GmbH, Edermünde, Germany). FFPE sections of 3 µm were cut, rehydrated and hematoxylin and eosin (H&E) stainings were performed (SAV liquid production GmbH, Flintsbach am Inn, Germany). The H&E sections were observed and analysed by an experienced neuropathologist (MM).

#### 4.3.2. Putative Cancer Stem Cell Markers

Tissue sections underwent an antigen retrieval step which was performed in a citrate buffer (pH 6) for nestin and in Tris/EDTA at 630 watts for Sox2 and CD95. After the unmasking step, the slides were left to cool down on ice for 20 min. For nestin, the Dako Envision+ system HRP rabbit kit (Dako) was used. In brief, after a blocking step of 10 min, the sections were incubated for 1 h with the antibody (Abcam ab176571, Cambridge, UK, 1.459 mg/mL, dilution 1:1000). After washing, the peroxidase-labelled polymer conjugated to goat anti-rabbit immunoglobulin was added for 30 min. Subsequently, the sections were incubated with DAB for 1 min and counterstained with hematoxylin (Merck). Finally, the samples were dehydrated in a series of increasing ethanol concentrations and the slides were mounted with Entallan (Merck, KGaA, Darmstadt, Germany). For Sox2 and CD95, the Dako Envision kit HRP rabbit was used. After a blocking step of 10 min, the sections were incubated for 1 h with the antibody (CD95: Cell Signaling Technologies 4233 S, Leiden, Netherlands, dilution 1:500; Sox2: Abcam ab133337, 0.108 mg/mL, dilution 1:250). After washing, the peroxidase-labelled polymer conjugated to goat anti-rabbit immunoglobulin was added for 30 min. Subsequently, the sections were incubated with DAB for 10 min and counterstained with hematoxylin (Merck). Finally, the samples were dehydrated in a series of increasing ethanol concentrations and the slides were mounted with Entallan (Merck, KGaA, Darmstadt, Germany). The Axioplan microscope (Zeiss, Germany) was used to observe the putative CSC markers’ stainings. The sections were visually inspected by two independent observers (SM, AL) to avoid subjective bias. The intensity of the staining was assessed, and a score ranging from 0 (negative) to 3 was attributed. The percentage of positivity in the tumour (0–100%) was evaluated.

### 4.4. DNA Isolation

Genomic DNA was extracted from 15–25 mg of fresh frozen tissue with the QIAamp DNA Tissue and Blood kit (Qiagen GmbH, Hilden, Germany) following the manufacturer´s protocol. In brief, the tissue was digested overnight at 56 °C with proteinase K. Then, ethanol and buffer AL were added to the solutions, and the mix was applied to the columns. After washing and drying, the DNA was eluted in 50–150 µL of elution buffer.

### 4.5. Microsatellite Analysis

Microsatellite analyses were performed with isolated DNA to verify the human origin of the xenogafts by the Institute for Legal Medicine of the Faculty of Medicine and University Hospital Carl Gustav Carus, Dresden (SH). After PCR amplification, 16 loci were analysed (D3S1358, vWA, D16S539, D2S1338, D8S1179, D21S11, D18S51, D19S433, TH01, FGA, D1S1656, D12S391, D10S1248, D22S1045, D2S441, the gender determination locus Amelogenin) with the ESSPlex kit (Qiagen) or the NGM kit (Thermofischer) according to the manufacturer’s instructions. The PCR products were analysed on ABI Prism^®^310 or 3500 Genetic Analyzer using the GeneMapper ID-X software (Applied Biosystems, Foster City, CA, USA).

### 4.6. Methylation Specific PCR

DNA isolated from fresh frozen tissue was converted with bisulfite using the Qiagen bisulfite conversion kit (Qiagen GmbH). For each sample, 500 ng of DNA was used for the conversion, along with water, bisulfite solution, and DNA protect buffer. The conversion was performed in a thermal cycler with the following conditions: 95 °C for 5 min, 60 °C for 20 min, 95 °C for 5 min, 60 °C for 20 min, 20 °C (hold) (1 cycle). The concentration of DNA was measured with the Qubit single-strand DNA protocol and the Qubit fluorometer (Invitrogen).

The used primers were designed according to [61,62]: 5’-TTTCGACGTTCGTAGGTTTTCGC-3’ (forward primer) and 5’-GCACTCTTCCGAAAACGAAACG-3’ (reverse primer) for methylated template detection (product length 81 bp) and 5’- TTTGTGTTTTGATGTTTGTAGGTTTTTGT-3’ (forward primer) and 5’-AACTCCACACTCTTCCAAAAACAAAACA-3’ (reverse primer) for unmethylated template detection (product length 93 bp) in the MGMT promoter. For the PCR, 150 ng of converted DNA was used. The following programme was used for the PCR: enzyme activation: 95 °C for 10 min; 40 cycles: 94 °C for 15 sec, 53 °C for 30 sec, 72 °C for 30 sec; finally, 72 °C for 10 min. For each PCR run, four controls provided by the manufacturer were used: converted methylated DNA, unmethylated DNA, genomic DNA, and a water control. PCR products were run on 2% agarose gel containing RedSafe staining solution (Intron Biotechnology, Kirkland, USA) to detect the DNA. The analysis was performed with ImageJ (Rasband, W.S., ImageJ, U. S. National Institutes of Health, Bethesda, Maryland, USA, https://imagej.nih.gov/ij/, 1997–2018) according to [61]. The optical signal for the detection of the methylated product (M) and unmethylated product (U) were quantified: Samples with M/U > 1 were considered to have a strongly methylated MGMT promoter; 0 <M/U< 1 were considered weakly methylated; and M/U = 0 unmethylated. In the case of all DK26 PDXs, DK33.4b and DK33.5, there was no amplification and signal for the non-methylation-specific PCR (U = 0); they were considered methylated.

### 4.7. Tumour Volume

To assess the tumour volumes, the length L (longest axis of the tumour) and the width W (perpendicular axis to the length) were measured weekly with a calliper (mm). Tumour volumes were calculated according to the ellipsoid volume formula (reviewed in [63]):

(1) $$Volume=\frac{\pi }{6}*L*W^{2}$$

The volume doubling time was estimated in the exponential growth phase. The latency time was defined as the time necessary to reach a tumour of 40 mm^3^ and calculated accordingly. In the models that were passaged for at least three generations, the cumulative growth curves were established according to [24]. The nlme package in R (R Foundation for Statistical Computing, Vienna, Austria, version 3.3.2) was used. Linear and quadratic models were fitted to the growth curves, and the coefficient were compared by an ANOVA procedure. The model with the lowest AIC (Akaike Information Criterion) was considered to be the best fit.

### 4.8. Statistical Analyses

Statistical analyses were performed with Excel (Microsoft, version 1908) and R (R Foundation for Statistical Computing, Vienna, Austria, version 3.3.2). For each experiment and each passage, the normal distribution of the data was verified with the Shapiro–Wilk test. Considering some data did not follow a normal distribution, the non-parametric Mann–Whitney test was performed for comparison of the growth data between passages. Correlations were assessed using the Spearman correlation coefficient (r). Graphics were made with the beeswarm package in R.

## 5. Conclusions

The present study describes the successful establishment of a panel of new preclinical glioblastoma PDXs. Although minor changes between primary material and PDXs have been observed, most of the models appear to have conserved the key characteristics of glioblastoma (CSC expression, histology, MGMT promoter methylation). Consequently, we propose that they would constitute suitable models to test new clinical treatment approaches and validate potential biomarkers.

## Figures and Tables

**Table 1:** Patient cohort for the glioblastoma patient-derived xenograft (PDX) study. ^1^

Cohort ID	Gender	Age	Methylation Status	Tumour Location
DK26	f	67	methylated	frontal
DK27	f	72	methylated	frontal
DK28	f	74	unmethylated	parieto-occipital
DK29	f	66	unmethylated	multi-ocular/fronto-parietal
DK30	m	57	methylated	opercular
DK32	m	66	unmethylated	parieto-occipital
DK33	f	73	methylated	frontal-temporal
DK34	f	82	methylated	parieto-occipital
DK35	f	75	unmethylated	parieto-occipital
DK36	m	85	unmethylated	frontal
DK37	m	79	unmethylated	temporal lobe
DK38	m	73	unmethylated	frontal
DK39	m	63	methylated	parieto-temporal to occipital
DK40	f	74	methylated	frontal lobe
DK41	m	53	unmethylated	frontal lobe
DK42	m	71	unmethylated	frontal lobe
DK45	m	79	unmethylated	temporal-occipital
DK51	m	67	methylated	frontal lobe
DK54	f	78	unmethylated	temporo-parietal
DK60	m	76	unmethylated	temporo-parietal
DK63	f	76	methylated	frontal
DK77	f	83	methylated	temporal-occipital

^1.^ Clinical information were obtained from the RadPlanBio database [23]. m: male (50%), f: female (50%). Methylation status corresponds to the methylation status of the *MGMT* promoter. Mean age: 72.2 years old. All patients were diagnosed with primary glioblastoma.

**Table 2:** Tumour take proportion of the primary material in the nude mice. ^2^

Experiment	Take Proportion	Number of Passages
DK26	7/10	3
DK28	2/10	1
DK29	7/10	5
DK30	2/10	2
DK32	1/10	1
DK33	7/10	3
DK35	1/5	2
DK39	5/10	3
DK41	5/10	1
DK42	3/10	1

^2.^ Tumour samples were transplanted to the axillae of five NMRI nude mice with 1–2 transplantation sites/mouse depending on the amount of primary material. Mice were monitored weekly and tumours were measured with a calliper. Growing tumours were excised and further transplanted to a mouse cohort to perpetuate the model. The take proportion indicates the number of growing tumours per transplantation site. Multiple tumours growing per transplantation site were not considered for the calculation of the take rate. The take rate at the different passages is shown in supplementary Table S1.

## Supplementary Materials

The following are available online at https://www.mdpi.com/2072-6694/12/4/871/s1, Table S1: Take in nude mice in the subsequent passages, Figure S1: Microsatellite analysis, Figure S2: Cumulative growth curve, Figure S3: Hematoxylin and eosin stainings of the patients’ tumour samples and the PDXs, Figure S4: Histological analysis of the tumours, Figure S5: Evaluation of Sox2 and nestin in the patients’ samples and in the PDXs, Figure S6: Evaluation of CD95 in the patients’ samples and in the PDXs.

## Abbreviations

**AIC**	Akaike Information Criterion
**CD95**	Cluster of Differentiation 95
**CDX**	Cell line derived xenograft
**CSC**	Cancer stem cell
**DK**	Deutsches Konsortium
**MRI**	Magnetic Resonance Imaging
**MGMT**	O6-MethylguaninDNA-Methyltransferase
**MSP**	Methylation-specific PCR
**M**	methylated template
**U**	unmethylated template
**PDX**	Patient-derived xenograft
**RT**	Radiotherapy
**Sox2**	Sex determining region Y-box 2
**VDT**	volume doubling time

## References

[1] Bray F., Ferlay J., Soerjomataram I., Siegel R.L., Torre L.A., Jemal A. (2018). Global cancer statistics 2018: GLOBOCAN estimates of incidence and mortality worldwide for 36 cancers in 185 countries. CA. Cancer J. Clin..

[2] Visser O., Ardanaz E., Botta L., Sant M., Tavilla A., Minicozzi P. (2015). Survival of adults with primary malignant brain tumours in Europe; Results of the EUROCARE-5 study. Eur. J. Cancer.

[3] Stupp R., Mason W.P., van den Bent M.J., Weller M., Fisher B., Taphoorn M.J.B., Belanger K., Brandes A.A., Marosi C., Bogdahn U. (2005). Radiotherapy plus Concomitant and Adjuvant Temozolomide for Glioblastoma. N. Engl. J. Med..

[4] Mann J., Ramakrishna R., Magge R., Wernicke A.G. (2018). Advances in radiotherapy for glioblastoma. Front. Neurol..

[5] Stupp R., Dietrich P.-Y., Kraljevic S.O., Pica A., Maillard I., Maeder P., Meuli R., Janzer R., Pizzolato G., Miralbell R. (2002). Promising survival for patients with newly diagnosed glioblastoma multiforme treated with concomitant radiation plus temozolomide followed by adjuvant temozolomide. J. Clin. Oncol..

[6] Rivera A.L., Pelloski C.E., Gilbert M.R., Colman H., De La Cruz C., Sulman E.P., Bekele B.N., Aldape K.D. (2010). MGMT promoter methylation is predictive of response to radiotherapy and prognostic in the absence of adjuvant alkylating chemotherapy for glioblastoma. Neuro Oncol..

[7] Singh S.K., Clarke I.D., Terasaki M., Bonn V.E., Hawkins C., Squire J., Dirks P.B. (2003). Identification of a cancer stem cell in human brain tumors. Cancer Res..

[8] Galli R., Binda E., Orfanelli U., Cipelletti B., Gritti A., Vitis S.D., Fiocco R., Foroni C., Dimeco F., Vescovi A. (2004). Isolation and characterization of tumorigenic, stem-like neural precursors from human glioblastoma. Cancer Res..

[9] Bao S., Wu Q., McLendon R.E., Hao Y., Shi Q., Hjelmeland A.B., Dewhirst M.W., Bigner D.D., Rich J.N. (2006). Glioma stem cells promote radioresistance by preferential activation of the DNA damage response. Nature.

[10] Baumann M., Krause M., Hill R. (2008). Exploring the role of cancer stem cells in radioresistance. Nat. Rev. Cancer.

[11] Bradshaw A., Wickremsekera A., Tan S.T., Peng L., Davis P.F., Itinteang T. (2016). Cancer stem cell hierarchy in glioblastoma multiforme. Front. Surg..

[12] Sathyan P., Zinn P.O., Marisetty A.L., Liu B., Kamal M.M., Singh S.K., Bady P., Lu L., Wani K.M., Veo B.L. (2015). Mir-21–Sox2 Axis Delineates Glioblastoma Subtypes with Prognostic Impact. J. Neurosci..

[13] Dahlstrand J., Collins V.P., Lendahl U. (1992). Expression of the class VI intermediate filament nestin in human central nervous system tumors. Cancer Res..

[14] Drachsler M., Kleber S., Mateos A., Volk K., Mohr N., Chen S., Cirovic B., Tüttenberg J., Gieffers C., Sykora J. (2016). CD95 maintains stem cell-like and non-classical EMT programs in primary human glioblastoma cells. Cell Death Dis..

[15] Kleber S., Sancho-Martinez I., Wiestler B., Beisel A., Gieffers C., Hill O., Thiemann M., Mueller W., Sykora J., Kuhn A. (2008). Yes and PI3K Bind CD95 to Signal Invasion of Glioblastoma. Cancer Cell.

[16] Taillandier L., Antunes L., Angioi-Duprez K.S. (2003). Models for neuro-oncological preclinical studies: Solid orthotopic and heterotopic grafts of human gliomas into nude mice. J. Neurosci. Methods.

[17] Kanabur P., Guo S., Simonds G.R., Kelly D.F., Gourdie R.G., Verbridge S.S., Sheng Z. (2016). Patient-derived glioblastoma stem cells respond differentially to targeted therapies. Patient-derived glioblastoma stem cells respond differentially to targeted therapies. Oncotarget.

[18] Joo K.M., Kim J., Jin J., Kim M., Seol H.J., Muradov J., Yang H., Choi Y.-L., Park W.-Y., Kong D.-S. (2013). Patient-specific orthotopic glioblastoma xenograft models recapitulate the histopathology and biology of human glioblastomas in situ. Cell Rep..

[19] Baumann M., DuBois W., Pu A., Freeman J., Suit H.D. (1992). Response of xenografts of human malignant gliomas and squamous cell carcinomas to fractionated irradiation. Int. J. Radiat. Oncol..

[20] Taghian A., Dubois W., Budach W., Baumann M., Freeman J., Suit H. (1995). In vivo radiation sensitivity of glioblastoma multiforme. Int. J. Radiat. Oncol..

[21] Sottoriva A., Spiteri I., Piccirillo S.G.M., Touloumis A., Collins V.P., Marioni J.C., Curtis C., Watts C., Tavaré S. (2013). Intratumor heterogeneity in human glioblastoma reflects cancer evolutionary dynamics. Proc. Natl. Acad. Sci. USA.

[22] Siolas D., Hannon G.J. (2013). Patient-derived tumor xenografts: Transforming clinical samples into mouse models. Cancer Res..

[23] Skripcak T., Belka C., Bosch W., Brink C., Brunner T., Budach V., Büttner D., Debus J., Dekker A., Grau C. (2014). Creating a data exchange strategy for radiotherapy research: Towards federated databases and anonymised public datasets. Radiother. Oncol..

[24] Pearson A.T., Finkel K.A., Warner K.A., Nör F., Tice D., Martins M.D., Jackson T.L., Nör J.E. (2016). Patient-derived xenograft (PDX) tumors increase growth rate with time. Oncotarget.

[25] Carlson B.L., Pokorny J.L., Schroeder M.A., Sarkaria J.N., Enna S.J., Williams M., Barret J.F., Ferkany J.W., Kenakin T., Porsolt R.D. (2011). Establishment, maintenance, and in vitro and in vivo applications of primary human glioblastoma multiforme (GBM) xenograft models for translational biology studies and drug discovery. Current Protocols in Pharmacology.

[26] William D., Mullins C.S., Schneider B., Orthmann A., Lamp N., Krohn M., Hoffmann A., Classen C.-F., Linnebacher M. (2017). Optimized creation of glioblastoma patient derived xenografts for use in preclinical studies. J. Transl. Med..

[27] Piccirillo S.G.M., Combi R., Cajola L., Patrizi A., Redaelli S., Bentivegna A., Baronchelli S., Maira G., Pollo B., Mangiola A. (2009). Distinct pools of cancer stem-like cells coexist within human glioblastomas and display different tumorigenicity and independent genomic evolution. Oncogene.

[28] Antunes L., Angioi-Duprez K.S., Bracard S.R., Klein-Monhoven N.A., Le Faou A.E., Duprez A.M., Plénat F.M. (2000). Analysis of tissue chimerism in nude mouse brain and abdominal xenograft models of human glioblastoma multiforme: What does it tell us about the models and about glioblastoma biology and therapy?. J. Histochem. Cytochem..

[29] Williams S.A., Anderson W.C., Santaguida M.T., Dylla S.J. (2013). Patient-derived xenografts, the cancer stem cell paradigm, and cancer pathobiology in the 21st century. Lab. Investig..

[30] Jung J., Seol H.S., Chang S. (2018). The generation and application of patient-derived xenograft model for cancer research. Cancer Res. Treat. Off. J. Korean Cancer Assoc..

[31] Quail D.F., Joyce J.A. (2017). The microenvironmental landscape of brain tumors. Cancer Cell.

[32] Ivanics T., Bergquist J.R., Liu G., Kim M.P., Kang Y., Katz M.H., Perez M.V.R., Thomas R.M., Fleming J.B., Truty M.J. (2018). Patient-derived xenograft cryopreservation and reanimation outcomes are dependent on cryoprotectant type. Lab. Investig. J. Tech. Methods Pathol..

[33] Guerrera F., Tabbò F., Bessone L., Maletta F., Gaudiano M., Ercole E., Annaratone L., Todaro M., Boita M., Filosso P.L. (2016). The influence of tissue ischemia time on RNA integrity and patient-derived xenografts (PDX) engraftment rate in a non-small cell lung cancer (NSCLC) biobank. PLoS ONE.

[34] Kageyama K., Ohara M., Saito K., Ozaki S., Terai M., Mastrangelo M.J., Fortina P., Aplin A.E., Sato T. (2017). Establishment of an orthotopic patient-derived xenograft mouse model using uveal melanoma hepatic metastasis. J. Transl. Med..

[35] Lathia J.D., Mack S.C., Mulkearns-Hubert E.E., Valentim C.L.L., Rich J.N. (2015). Cancer stem cells in glioblastoma. Genes Dev..

[36] Schmitz M., Temme A., Senner V., Ebner R., Schwind S., Stevanovic S., Wehner R., Schackert G., Schackert H.K., Fussel M. (2007). Identification of SOX2 as a novel glioma-associated antigen and potential target for T cell-based immunotherapy. Br. J. Cancer.

[37] Berezovsky A.D., Poisson L.M., Cherba D., Webb C.P., Transou A.D., Lemke N.W., Hong X., Hasselbach L.A., Irtenkauf S.M., Mikkelsen T. (2014). Sox2 Promotes Malignancy in Glioblastoma by Regulating Plasticity and Astrocytic Differentiation. Neoplasia.

[38] Strojnik T., Røsland G.V., Sakariassen P.O., Kavalar R., Lah T. (2007). Neural stem cell markers, nestin and musashi proteins, in the progression of human glioma: Correlation of nestin with prognosis of patient survival. Surg. Neurol..

[39] Lin A. (2015). Role of nestin in glioma invasion. World J. Transl. Med..

[40] Lv D., Lu L., Hu Z., Fei Z., Liu M., Wei L., Xu J. (2017). Nestin expression is associated with poor clinicopathological features and prognosis in glioma patients: An association study and meta-analysis. Mol. Neurobiol..

[41] Chinnaiyan P., Wang M., Rojiani A.M., Tofilon P.J., Chakravarti A., Ang K.K., Zhang H.-Z., Hammond E., Curran W., Mehta M.P. (2008). The prognostic value of nestin expression in newly diagnosed glioblastoma: Report from the Radiation Therapy Oncology Group. Radiat. Oncol. Lond. Engl..

[42] Lu W.J., Lan F., He Q., Lee A., Tang C.Z., Dong L., Lan B., Ma X., Wu J.C., Shen L. (2011). Inducible expression of stem cell associated intermediate filament nestin reveals an important role in glioblastoma carcinogenesis. Int. J. Cancer.

[43] Morgan K.M., Riedlinger G.M., Rosenfeld J., Ganesan S., Pine S.R. (2017). Patient-derived xenograft models of non-small cell lung cancer and their potential utility in personalized medicine. Front. Oncol..

[44] Sarkaria J.N., Hu L.S., Parney I.F., Pafundi D.H., Brinkmann D.H., Laack N.N., Giannini C., Burns T.C., Kizilbash S.H., Laramy J.K. (2018). Is the blood–brain barrier really disrupted in all glioblastomas? A critical assessment of existing clinical data. Neuro Oncol..

[45] van Tellingen O., Yetkin-Arik B., de Gooijer M.C., Wesseling P., Wurdinger T., de Vries H.E. (2015). Overcoming the blood–brain tumor barrier for effective glioblastoma treatment. Drug Resist. Updat..

[46] Yaromina A., Krause M., Thames H., Rosner A., Krause M., Hessel F., Grenman R., Zips D., Baumann M. (2007). Pre-treatment number of clonogenic cells and their radiosensitivity are major determinants of local tumour control after fractionated irradiation. Radiother. Oncol..

[47] Gurtner K., Hessel F., Eicheler W., Dörfler A., Zips D., Heider K.-H., Krause M., Baumann M. (2012). Combined treatment of the immunoconjugate bivatuzumab mertansine and fractionated irradiation improves local tumour control in vivo. Radiother. Oncol. J. Eur. Soc. Ther. Radiol. Oncol..

[48] Fichtner I., Rolff J., Soong R., Hoffmann J., Hammer S., Sommer A., Becker M., Merk J. (2008). Establishment of patient-derived non–small cell lung cancer xenografts as models for the identification of predictive biomarkers. Clin. Cancer Res..

[49] Zhao S.G., Yu M., Spratt D.E., Chang S.L., Feng F.Y., Kim M.M., Speers C.W., Carlson B.L., Mladek A.C., Lawrence T.S. (2019). Xenograft-based, platform-independent gene signatures to predict response to alkylating chemotherapy, radiation, and combination therapy for glioblastoma. Neuro Oncol..

[50] DeRose Y.S., Wang G., Lin Y.-C., Bernard P.S., Buys S.S., Ebbert M.T.W., Factor R., Matsen C., Milash B.A., Nelson E. (2011). Tumor grafts derived from women with breast cancer authentically reflect tumor pathology, growth, metastasis and disease outcomes. Nat. Med..

[51] Ben-David U., Ha G., Tseng Y.-Y., Greenwald N.F., Oh C., Shih J., McFarland J.M., Wong B., Boehm J.S., Beroukhim R. (2017). Patient-derived xenografts undergo murine-specific tumor evolution. Nat. Genet..

[52] Peng S., Creighton C.J., Zhang Y., Sen B., Mazumdar T., Myers J.N., Woolfson A., Lorenzi M.V., Bell D., Williams M.D. (2013). Tumor grafts derived from patients with head and neck squamous carcinoma authentically maintain the molecular and histologic characteristics of human cancers. J. Transl. Med..

[53] Vaubel R.A., Tian S., Remonde D., Schroeder M.A., Mladek A.C., Kitange G.J., Caron A., Kollmeyer T.M., Grove R., Peng S. (2019). Genomic and phenotypic characterization of a broad panel of patient derived xenografts reflects the diversity of glioblastoma. Clin. Cancer Res..

[54] Zhang X., Claerhout S., Prat A., Dobrolecki L.E., Petrovic I., Lai Q., Landis M.D., Wiechmann L., Schiff R., Giuliano M. (2013). A Renewable tissue resource of phenotypically stable, biologically and ethnically diverse, patient-derived human breast cancer xenograft models. Cancer Res..

[55] Braekeveldt N., Von Stedingk K., Fransson S., Martinez-Monleon A., Lindgren D., Axelson H., Levander F., Willforss J., Hansson K., Øra I. (2018). Patient-derived xenograft models reveal intratumor heterogeneity and temporal stability in neuroblastoma. Cancer Res..

[56] Bruna A., Rueda O.M., Greenwood W., Batra A.S., Callari M., Batra R.N., Pogrebniak K., Sandoval J., Cassidy J.W., Tufegdzic-Vidakovic A. (2016). A biobank of breast cancer explants with preserved intra-tumor heterogeneity to screen anticancer compounds. Cell.

[57] Eirew P., Steif A., Khattra J., Ha G., Yap D., Farahani H., Gelmon K., Chia S., Mar C., Wan A. (2015). Dynamics of genomic clones in breast cancer patient xenografts at single cell resolution. Nature.

[58] Smiraglia D.J., Rush L.J., Frühwald M.C., Dai Z., Held W.A., Costello J.F., Lang J.C., Eng C., Li B., Wright F.A. (2001). Excessive CpG island hypermethylation in cancer cell lines versus primary human malignancies. Hum. Mol. Genet..

[59] Yamada H., Vijayachandra K., Penner C., Glick A. (2001). Increased sensitivity of transforming growth factor (TGF) β1 null cells to alkylating agents reveals a novel link between TGFβ signaling and O 6-methylguanine methyltransferase promoter hypermethylation. J. Biol. Chem..

[60] Danam R.P., Howell S.R., Remack J.S., Brent T.P. (2001). Heterogeneous methylation of the O6-methylguanine-DNA methyltransferase promoter in immortalized IMR90 cell lines. Int. J. Oncol..

[61] Esteller M., Garcia-Foncillas J., Andion E., Goodman S.N., Hidalgo O.F., Vanaclocha V., Baylin S.B., Herman J.G. (2000). Inactivation of the DNA-repair gene MGMT and the clinical response of gliomas to alkylating agents. N. Engl. J. Med..

[62] Christians A., Hartmann C., Benner A., Meyer J., von Deimling A., Weller M., Wick W., Weiler M. (2012). Prognostic value of three different methods of mgmt promoter methylation analysis in a prospective trial on newly diagnosed glioblastoma. PLoS ONE.

[63] Tomayko M.M., Reynolds C.P. (1989). Determination of subcutaneous tumor size in athymic (nude) mice. Cancer Chemother. Pharmacol..

