# Supplementary material for: Establishment and Characterisation of Heterotopic Patient-Derived Xenografts for Glioblastoma
Source: Cancers (Basel). 2020 Apr 3;12(4):871. doi: 10.3390/cancers12040871 (PMC7226316; doi:10.3390/cancers12040871)
Supplement: Supplementary file 1 [file cancers-12-00871-s001.pdf]

Supplementary Materials

Establishment and Characterisation of Heterotopic Patient-Derived Xenografts for Glioblastoma

Sarah Meneceur, Annett Linge, Matthias Meinhardt, Sandra Hering, Steffen Löck, Rebecca Bütof, Dietmar Krex, Gabriele Schackert, Achim Temme, Michael Baumann, Mechthild Krause and Cläre von Neubeck

Table S1. Take in nude mice in the subsequent passages.

Experiment	Take rate Passage 1	Number of passage	Take rate in the subsequent passages			
			2	3	4	5
DK26	70%	3	57%	100%		
DK28	20%	1				
DK29	70%	5	60%	10%	30%	90%
DK30	20%	2	63%			
DK32	10%	1				
DK33	70%	3	93%	83%		
DK35	20%	2	0 (0/5)			
DK39	50%	3	30%	80%		
DK41	50%	1				
DK42	30%	1				

Tumour samples were transplanted in the axilla of NMRI nude mice with 1-2 transplantation sites depending on the amount of material. Mice were monitored weekly and tumors were measured with a caliper. Growing tumours were excised and further transplanted to a mouse cohort to perpetuate the model. Multiple tumours growing per transplantation site were not considered for the calculation of the take rate.

A)

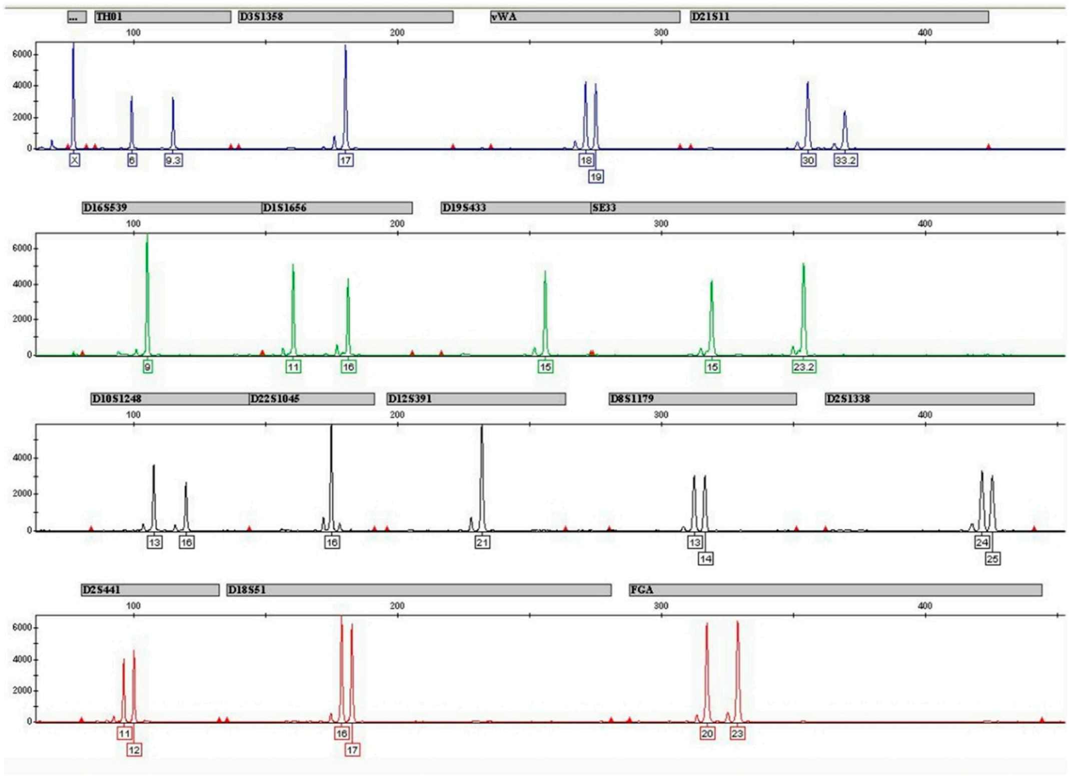

B)

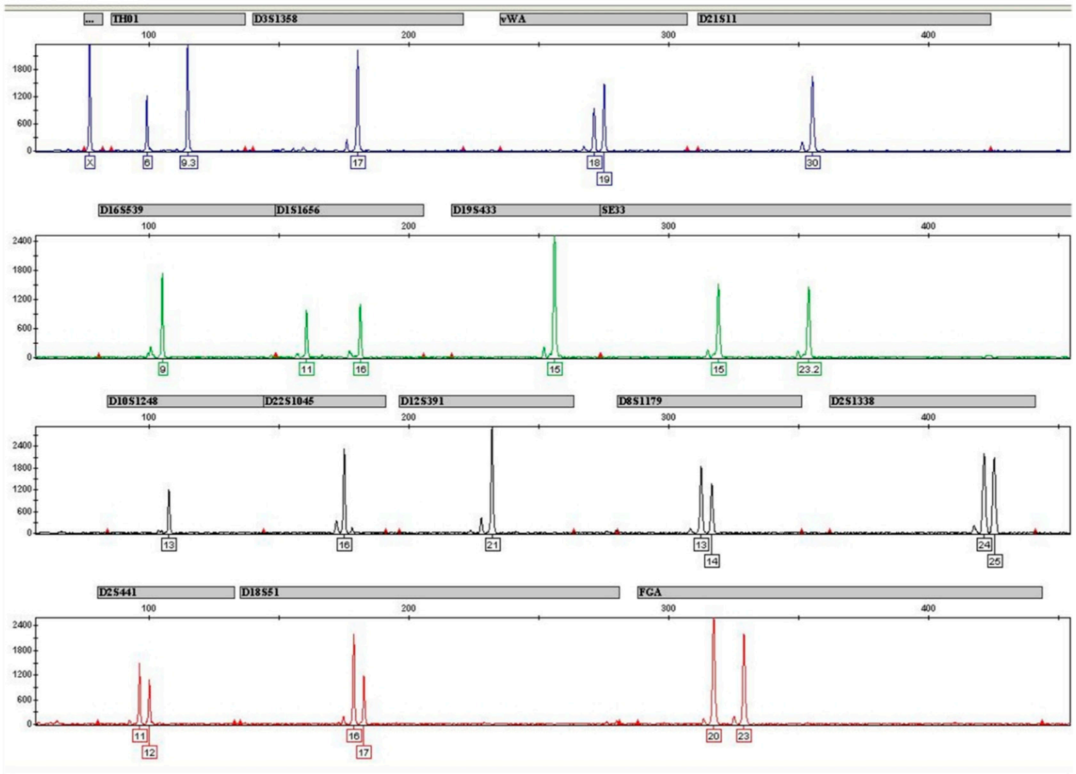

Figure S1. Microsatellite analysis in (**A**) the patients' samples DK33 and (**B**) one of the deriving PDX. The microsatellite profile is similar between the patient's sample and the PDX. Loss of heterozygosity can be observed for selected loci.

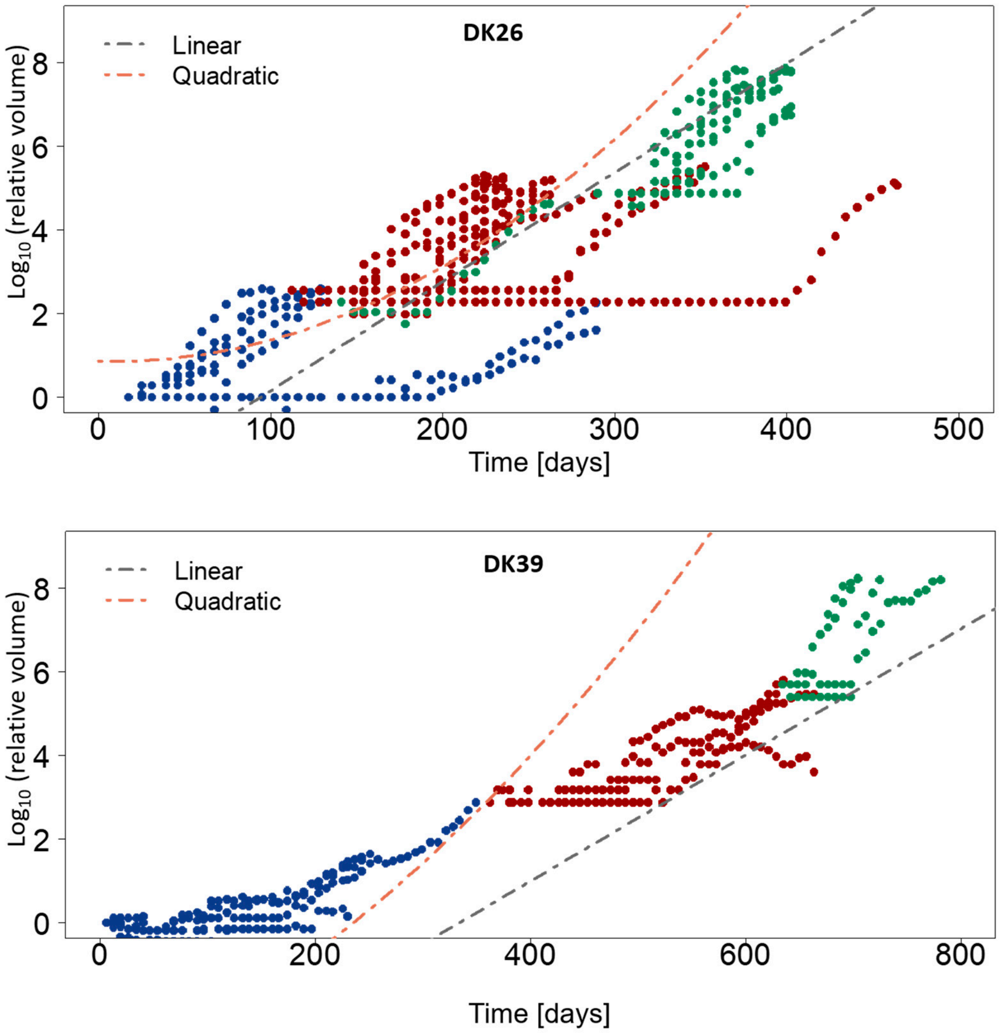

Figure S2. Cumulative growth curve in (**A**) DK26 and (**B**) DK39. The linear and quadratic model fit are represented as dotted lines. Each dot represents a tumour measurement of a growing tumour. Blue: passage 1; red: passage 2; green: passage 3.

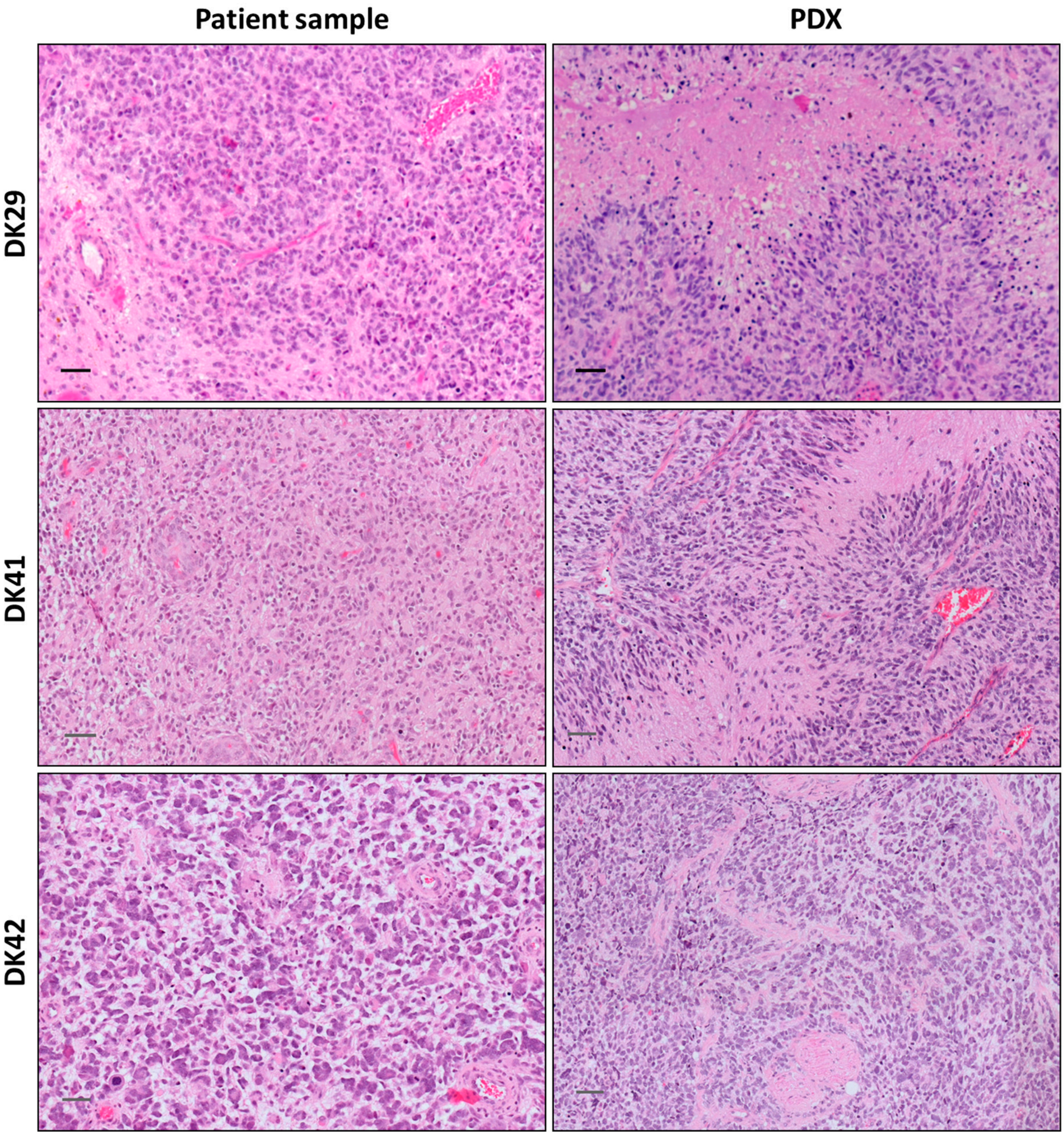

Figure S3. Hematoxylin and eosin stainings of the patients' samples and the PDX. Scale bar = 50 µm. The PDX present glioblastoma histological characteristics: pleomorphism, vascular proliferation, pseudopalisading.

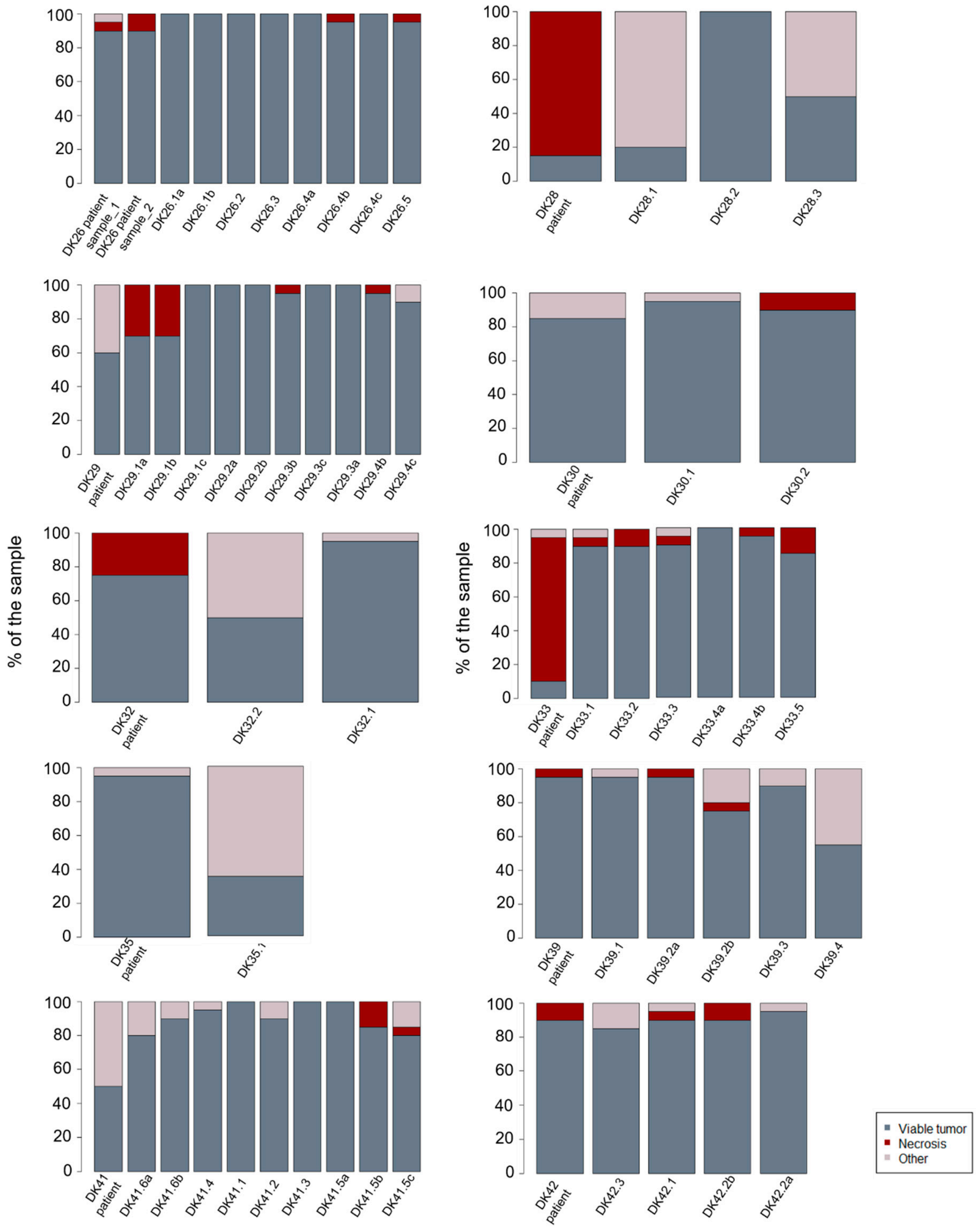

Figure S4. Histological analysis of the tumours. The histology of the respective patient material was compared to the deriving PDX similarity and was assessed as high for DK26, DK29, DK30, DK32, DK41 and DK42; medium for DK39; low for DK35 (DK28 and DK33 could not be assessed).

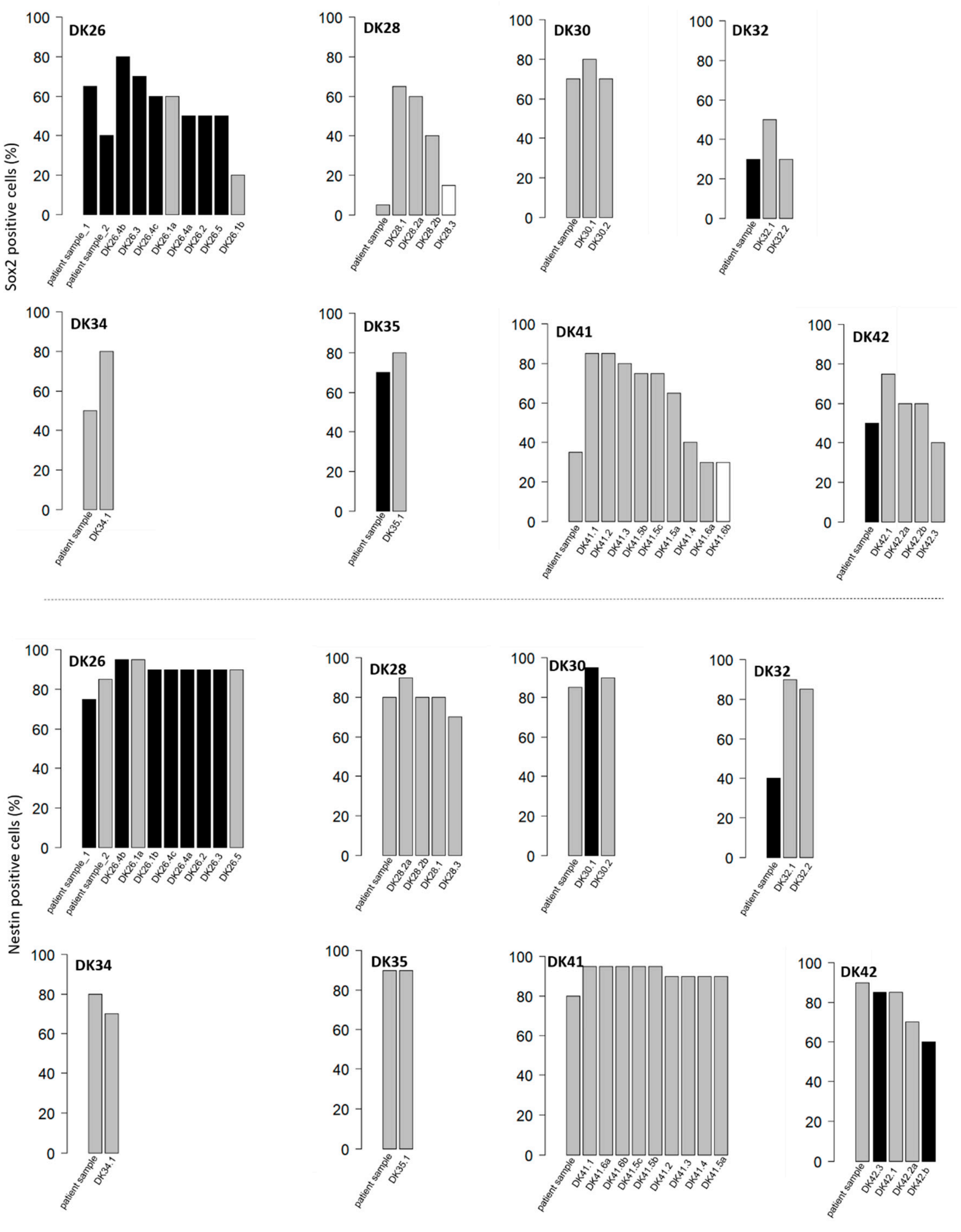

Figure S5. Evaluation of sox2 and nestin in the patients' samples and in the PDX. The stainings were evaluated by two independent observers, the proportion of positive cells (0–100%) and the intensity of the staining (1–3) were assessed (white: intensity score 1, grey: intensity score 2, black: intensity score 3).

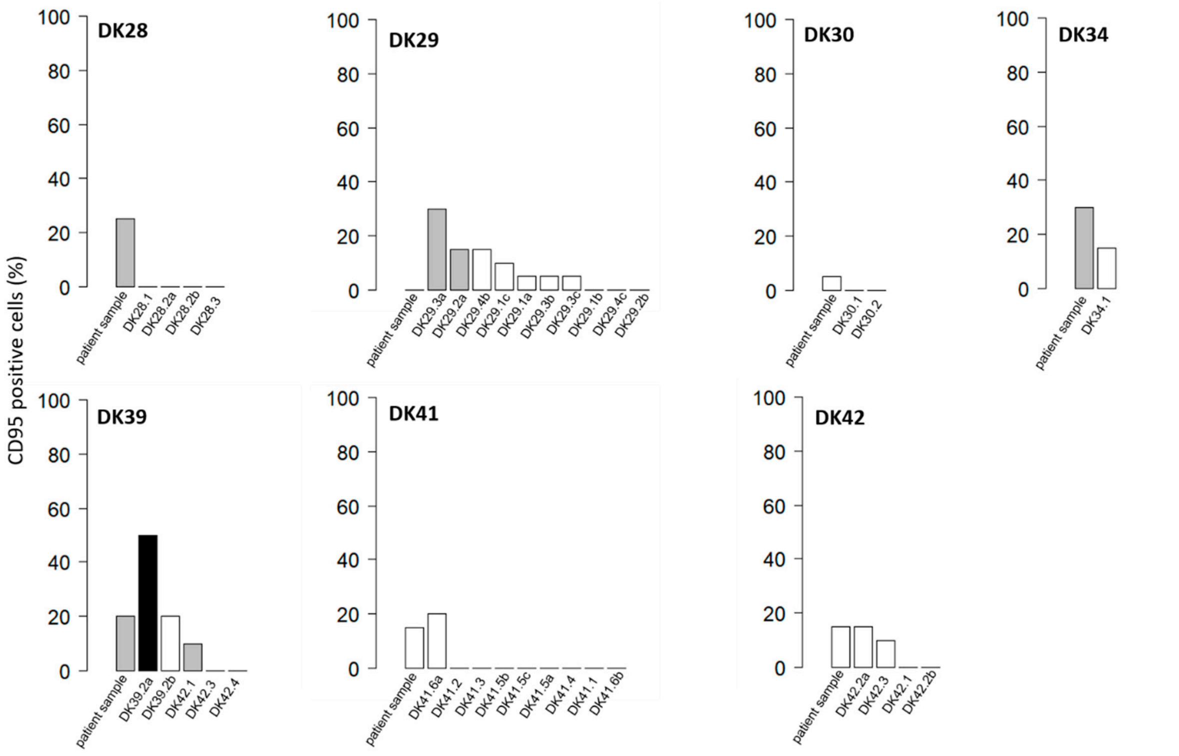

Figure S6. Evaluation of CD95 in the patients' samples and in the PDX. The stainings were evaluated by two independent observers, the proportion of positive cells (0–100%) and the intensity of the staining (1–3) were assessed (white: intensity score 1, grey: intensity score 2, black: intensity score 3).

© 2020 by the authors. Licensee MDPI, Basel, Switzerland. This article is an open access article distributed under the terms and conditions of the Creative Commons Attribution (CC BY) license (http://creativecommons.org/licenses/by/4.0/).
